# Sheep Welfare in Confined and Pasture Production Systems: A Comparative Study with Emphasis on Parasitological Status

**DOI:** 10.3390/vetsci13060589

**Published:** 2026-06-18

**Authors:** Katarina Nenadović, Dejan Bugarski, Tamara Ilić

**Affiliations:** 1 Department of Animal Hygiene, Faculty of Veterinary Medicine, University of Belgrade, Bulevar Oslobodjenja 18, 11000 Belgrade, Serbia; 2 Scientific Veterinary Institute “Novi Sad”, Rumenački put 20, 21000 Novi Sad, Serbia; dejan@niv.ns.ac.rs; 3 Department of Parasitology, Faculty of Veterinary Medicine, University of Belgrade, Bulevar Oslobodjenja 18, 11000 Belgrade, Serbia; tamara@vet.bg.ac.rs

**Keywords:** sheep welfare, AWIN protocol for sheep, confined farming, pasture farming, parasitic infections

## Abstract

**Background:** While traditional pasture-based systems offer sheep natural living conditions and freedom, barn farming provides greater environmental control and protection. The choice of farming system is a crucial factor directly affecting the physical and mental health of the animals. **Aim:** This study evaluated welfare indicators and parasite prevalence in different sheep management systems, examining the impact of parasitic infections on ewe welfare. **Methods:** Conducted from November 2025 to April 2026 across 26 farms (13 confined and 13 pasture), the study individually assessed a sample of 1192 ewes aged 2–7 years. Animal-based welfare indicators were assessed using the AWIN protocol for sheep, while parasitic infections were determined from fecal sampling. **Results:** The results revealed significant differences (*p* < 0.001) between the systems. Pasture farms showed a higher prevalence of soiled fleece (60.14%), skin lesions, ocular and nasal discharge (6.35%; 31.22%), respiratory issues (16.75%), fecal soiling (16.23%), borderline anemia (16.23%), and excessive itching (16.23%). In contrast, confined systems exhibited higher prevalence of fleece loss (36.00%), hoof overgrowth (20.96%), udder asymmetry (1.44%), and wool pulling (8.32%). Identified parasites included *Eimeria* spp., gastrointestinal strongyles, *Trichuris ovis*, *Dictyocaulus filarial*, *Muellerius capillaris*, *Protostrongylus rufescens*, *Moniezia* spp., *Dicrocoelium dendriticum*, *Fasciola hepatica*, *Paramphistomum* spp., and *Giardia intestinalis.* Significant correlations (*p* < 0.001) were found between certain welfare indicators and parasite infections in confined systems, such as between thin body condition score and *Eimeria* spp., and between fleece quality and fecal soiling with gastrointestinal strongyles, while in pasture systems, thin BCS, nasal discharge, and respiration quality correlated with *Muellerius capillaris*, borderline anemia with *Trichuris ovis* and *Dictyocaulus filaria*, and fecal soiling with *Eimeria* spp. and *Dicrocoelium dendriticum*. **Conclusions:** This data underscores the critical need to improve ewe welfare and implement targeted parasite control strategies in both farming systems.

## Simple Summary

This study evaluated the effects of different farming systems on the welfare and parasite prevalence of 1192 ewes across 26 farms from November 2025 to April 2026. The research compared confined indoor housing with pasture-based systems. The results revealed significant welfare challenges in both environments. Ewes in pasture systems suffered more frequently from soiled fleece, skin lesions, ocular and nasal discharge, respiratory issues, fecal soiling, borderline anemia, and excessive itching. In contrast, confined systems showed higher prevalence of fleece loss, hoof overgrowth, udder asymmetry, and wool pulling. Additionally, identified parasites included *Eimeria* spp., gastrointestinal strongyles, *Trichuris ovis*, *Dictyocaulus filaria*, *Muellerius capillaris*, *Protostrongylus rufescens*, *Moniezia* spp., *Dicrocoelium dendriticum*, *Fasciola hepatica*, *Paramphistomum* spp., and *Giardia intestinalis.* The study found clear correlations between specific parasitic infections and poor welfare indicators. This data underscores the critical need to improve ewe welfare and implement targeted parasite control strategies in both farming systems.

## 1. Introduction

Animal welfare refers to a state of complete physical, mental, and behavioral well-being, and it largely depends on an animal’s ability to adapt to changes in its external environment [1]. The ability of an individual animal to effectively cope with and adapt to its environment is a key component of animal welfare [2]. For this reason, animal welfare has become increasingly important in modern livestock production, where it is recognized as a key factor influencing health, reproduction, and product quality.

In sheep farming, the choice of production system plays a crucial role in ensuring appropriate living conditions. The two most commonly used systems for sheep are traditional pasture-based farming (extensive system) and barn farming (intensive system) [3]. The extensive system allows sheep to remain in open areas, where they can move freely, graze, and engage in social interactions, which positively affects their natural behavior and reduces stress levels [4]. However, despite being considered the more animal-friendly housing system, it can still present serious risks to animal health and welfare, such as dependence on weather conditions, variations in feed quality, and increased exposure to diseases, parasites, and predators [3]. Grazing sheep often encounter multiple parasites, including gastrointestinal nematodes, tapeworms, liver flukes, protozoa, and ectoparasites, making it essential to understand the species present in specific farming areas to develop effective control and treatment strategies [5]. Nematodes and liver flukes are particularly significant, as they are the primary contributors to productivity losses. According to many authors, gastrointestinal parasites can affect animal production, health, and welfare by causing discomfort, pain, and suffering, as well as both subclinical and clinical diseases [6,7]. In severe or prolonged cases, these infections can even lead to death, resulting in significant economic losses in grazing systems [8].

On the other hand, the intensive system provides better control over nutrition, health management, and reproduction. Nevertheless, limited space and restricted opportunities to express natural behaviors may lead to stress, frustration, and behavioral disorders [9]. Additionally, higher stocking densities increase the risk of disease transmission, and maintenance costs are significantly higher [10].

Although both systems have their advantages and disadvantages, it is clear that sheep welfare depends on multiple factors, including nutrition, environmental conditions, health care, and overall management. Therefore, a combination of these systems is often applied in practice to achieve optimal living conditions while maintaining efficient production.

The study aimed to evaluate ewe welfare and parasite infections in both confined and pasture farming systems. Understanding these relationships is highly significant, as it addresses a critical gap in targeted animal management by identifying how specific housing stressors interact with parasitic burdens to compromise sheep welfare. Integrating animal welfare assessment with parasitological monitoring could allow for the early detection of parasitic infections and serve as a basis for developing more effective management and parasite control strategies to improve sheep health, welfare, and productive performance.

## 2. Materials and Methods

### 2.1. Animals and Husbandry

The study was conducted between November 2025 and April 2026. Data were collected from a total of 26 sheep farms, including 13 confined and 13 pasture systems. The confined farms had an average of 207.5 ± 69.70 animals, while the pasture farms maintained an average of 171.2 ± 44.68 animals. The primary purpose of these farms was to rear lambs for meat production. The Romanov, Württemberg, Suffolk, and Île de France sheep breeds were raised on intensive farms, while the Vlasicka Pramenka breed prevailed on pasture farms. The examined intensive farms are located in the Srem region of Vojvodina, Serbia, whereas pasture production systems are predominantly distributed along the slopes of Fruška Gora Mountain, where flocks are provided with access to natural pastures. Serbia is located in Southeastern Europe, between 41°53′ and 46°11′ N latitude and 18°49′ and 23°00′ E longitude in the central part of the Balkan Peninsula, where a temperate continental climate predominates, with pronounced seasonal variations. Vojvodina occupies the northern part of Serbia and lies predominantly within the Pannonian Plain. The climate of Vojvodina is characterized by warm summers, moderately cold winters, and relatively evenly distributed precipitation throughout the year. The average annual air temperature ranges from 10 to 12 °C, while annual precipitation varies between 550 and 700 mm. The Srem District is situated in the southwestern part of Vojvodina, between the Sava and Danube rivers. The area is characterized by a combination of lowland terrain and the gently elevated slopes of the Fruška Gora Mountain. These geographical features contribute to the formation of favorable microclimatic conditions. During the vegetation period, favorable temperature and humidity conditions prevail, enabling the successful development, survival, and spread of various parasite species.

Ewes in the confined production system were managed under a total confinement housing strategy. These animals were kept indoors continuously throughout the entire study period, with zero access to pastures or outdoor exercise areas. The housing facilities featured concrete flooring bedded with straw, which was periodically renewed, and relied entirely on natural ventilation through window openings. Feeding and watering were managed entirely indoors using standardized troughs and automatic drinkers. Nutritional requirements were met through controlled feeding regimes, while water was provided ad libitum. On most farms operating under a confined system, sheep are feed twice a day with maize grain (administered at an average range of 150–400 g/head/day, depending on the physiological stage), wheat or barley feed, alfalfa hay (*Medicago sativa*), and soybean meal. Alfalfa hay is preferred by local farmers due to its high natural abundance and widespread cultivation in this region, making it highly accessible and cost-effective forage. Within their feeding strategy, it provides excellent nutritional value, particularly high crude protein, while ensuring favorable agronomic yields that reduce overall feed production costs. Reproductive and health management practices are fully implemented under human supervision. Deworming is carried out twice a year using ivermectin, as well as hoof trimming. Within the pasture system, management and grazing practices are flexible and highly dependent on seasonal weather conditions. During mild winters without snow cover, ewes remain on the pastures throughout the entire winter period. However, in the event of heavy snowfall or severe winter weather, the flocks are moved to traditional barns, where they receive supplementary indoor feeding consisting of alfalfa hay (*Medicago sativa*) and maize grain (administered at an average range of 100–200 g/head/day). During the grazing period, the primary water supply for the animals is provided by natural springs or streams available on the pastures, which may provide suitable habitats and optimal ecological conditions for snails of the family Lymnaeidae. Protection from adverse climatic conditions during active grazing is provided by dense vegetation cover, including trees, shrubs, and other vegetation, which contributes to the mitigation of environmental stressors. Deworming is performed once or twice a year on most farms using albendazole and levamisole, although some farms do not administer antiparasitic treatments.

### 2.2. Assessment of Animal Welfare

A total of 625 ewes from confined farms and 567 ewes from pasture farms, aged between 2 and 7 years, were included in the observations and assessed individually. The sample size for the ewe population was determined based on a power analysis, assuming a 50% prevalence of the trait under investigation (which requires the largest possible sample size for binomial traits), with a confidence level of 95% and a precision of ±10% [11]. The welfare assessment was conducted between 09:00 h and 16:00 h by two assessors, using the AWIN welfare assessment protocol for sheep [11]. For the pasture production systems, all individual assessments were carried out directly on the pasture. To ensure standard diagnostic accuracy, the animals were temporarily slowed down and restrained on-site by utilizing natural landscape barriers (such as dense bushes and vegetation) with the assistance of shepherds. This standardized framework is structured around four welfare principles, which are further divided into twelve criteria. Specifically, the principle of Good Feeding encompasses the criteria of prolonged hunger and prolonged thirst, while Good Housing addresses comfort around resting, thermal comfort, and ease of movement. Furthermore, the principle of Good Health includes the criteria of absence of injuries, absence of disease, and absence of pain induced by management procedures. Finally, Appropriate Behavior is evaluated through the criteria of social behavior expression, other behavior expression, a good human–animal relationship, and a positive emotional state. To address these criteria within the scope of this study, we selected specific animal-based indicators, including body condition score (BCS), fleece cleanliness, body and head lesions, leg injuries, lameness, fecal soiling, ocular and nasal discharge, respiration quality, mucosa color, mastitis and udder lesions, udder asymmetry, fleece quality, hoof overgrowth, excessive itching, and wool pulling—stereotypic behavior. Welfare indicators were scored 0 when welfare was good or 1 when welfare was poor and unacceptable. A higher score represented impaired sheep welfare. For body condition assessment, we used the BCS system with a scale from 0 to 5 described by Russell et al. [12], i.e., 1—emaciated, 2—thin, 3—average, 4—fat, and 5—obese conditions. Fleece cleanliness is scored a five-point scale, i.e., 0—clean and dry, 1—dry or damp, light soiling, 2—wet, soiled with mud or feces, 3—very wet, heavily soiled, and 4—filthy. Lesions on the head, neck, legs, and udder scored as 0—absence; 1—presence of the welfare indicator. Udder asymmetry was assessed in accordance with the AWIN protocol to detect potential chronic mastitis or tissue atrophy. Due to the study timeframe (November–April), ewes were evaluated across mixed production stages (late gestation, lactation, and post-weaning). The assessment was strictly visual, performed from behind while ewes were standing or moving slowly, without manual palpation. Although the standard AWIN protocol focuses on housed animals for hoof overgrowth, this indicator was evaluated in both housing systems to ensure a comprehensive assessment. The evaluation was conducted strictly visually without manual restraint to avoid handling stress, by performing inspections while animals stood or walked slowly on firm, flat ground, scored as 0—absence and 1—presence of hoof overgrowth. Lameness was scored on four levels, i.e., 0—not lame, 1—minor lameness, 2—lame, 4—severe lameness. Fecal soiling is the presence of fecal material on the wool around the anus, breech area, tail and hind legs, scored as 0—not present, 1—very light soiling, 2—light soiling and dags, 3—soiling and dags, and 4—extensive soiling and dags. Mucosa color was scored as 0—not anemic, 1—not anemic, 2—borderline anemia, 3—anemic, and 4—severely anemic. Fleece quality was evaluated on three levels, i.e., 0—fleece quality good, 1—some fleece loss, and 2—significant fleece loss. To ensure a methodologically sound evaluation and to prevent observer induced behavioral modifications, the assessment of abnormal behavior was strictly separated from the individual handling. Wool pulling was recorded using a scan sampling method by visually observing the undisturbed flock from a distance for a prolonged period before close individual inspection, ensuring that the animals were not aware of or altered by the presence of the observers. Finally, the approximate cumulative time of 8 min per ewe represents strictly the total duration required to complete the entire sequence of individual visual and clinical assessments in accordance with the standard AWIN protocol guidelines for sheep.

### 2.3. Sample Collection and Parasitological Analyses

Parasitological examinations were performed at the Department of Parasitology, University of Belgrade Faculty of Veterinary Medicine, on fecal samples of sheep. Coprological testing included both macroscopic and microscopic examinations of samples. Individual samples were collected from the same housing unit, regarding housing systems.

#### 2.3.1. Sample Collections

Fecal samples were collected from all sheep (1192 animals) that underwent welfare assessment. Individual fecal samples were obtained directly from the rectum of each animal to avoid environmental contamination. In pasture systems, samples were collected while animals were at pasture, whereas in confined systems, sampling was conducted within housing facilities. To minimize diurnal variation in parasite egg shedding, all samples were collected in the morning hours, between 07:00 and 10:00 h, before major feeding and/or turnout to pasture. Fresh fecal samples were placed in labeled sterile containers, identified by farm and animal code, and transported to the laboratory in cooled conditions (4 °C) within 24 h of collection. Parasitological examination was performed immediately upon arrival using standard coprological techniques for detection and identification of helminth and protozoan stages.

#### 2.3.2. Macroscopic Examination

In the macroscopic examination, the formation, consistency, color, and odor of fecal samples were investigated. Any deviations in these parameters from the typical physiological characteristics of the feces of the sheep were noted. The presence of impurities such as blood, pus, mucus, or undigested food was recorded as potential markers of certain pathological conditions of the gastrointestinal tract. Thereafter, the feces was carefully examined using tweezers, and any adult helminths and their parts were transferred to a Petri dish, rinsed in saline, and prepared for further analysis [13].

#### 2.3.3. Microscopic Examination

Fecal samples were examined using qualitative and quantitative coprological techniques for the detection, identification, and quantification of gastrointestinal, pulmonary, and hepatobiliary parasites.

#### 2.3.4. Qualitative Microscopic Examination

Microscopic diagnosis was performed using both direct (non-concentration) and concentration techniques. Direct wet mount examination was carried out using the Vajda method for rapid screening of fresh fecal material. Concentration of parasitic elements was achieved using flotation and sedimentation techniques to improve detection sensitivity [14]. Flotation was performed using a saturated sodium chloride (NaCl) solution (>97%; Roth, Karlsruhe, Germany), prepared by dissolving 210 g of NaCl in 1000 mL of distilled water (specific gravity 1.200). Sedimentation techniques were applied for the detection of heavier eggs and trematode structures that do not float in saturated solutions.

#### 2.3.5. Quantitative Examination and LOD Interpretation

Quantification of parasitic burden was performed using the McMaster technique [15] with a sensitivity (limit of detection, LOD) of 50 eggs/oocysts/cysts per gram (EPG/OPG) of feces [16]. Results were expressed as follows:

- EPG (eggs per gram) for helminths.
- OPG (oocysts per gram) for coccidia (*Eimeria* spp.).
- CPG (cysts per gram) for protozoa (*Giardia intestinalis*).

The following interpretative thresholds were applied for infection intensity:

- Low infection: 50–500 EPG/OPG.
- Moderate infection: 550–1500 EPG/OPG.
- High infection: >1500 EPG/OPG.

For *Eimeria* spp., values > 5000 OPG were considered indicative of clinically relevant coccidial infection in adult ewes. For gastrointestinal strongyles, EPG values > 500 were considered suggestive of subclinical infection, while values > 1000 EPG were interpreted as potentially clinically significant depending on the host condition and management system. For *Fasciola hepatica* and *Dicrocoelium dendriticum*, detection of eggs was considered diagnostically significant regardless of EPG level, due to the poor correlation between egg output and infection severity in chronic trematode infections.

Microscopic examination was performed under 100× magnification for morphological identification of parasitic stages, including the following:

- Nematode eggs (strongyle-type eggs of gastrointestinal nematodes, *Trichuris ovis*).
- Cestode eggs (*Moniezia* spp.).
- Trematode eggs (*F. hepatica*, *D. dendriticum*, *Paramphistomum* spp.).
- Protozoan oocysts (*Eimeria* spp.) and cysts (*G. intestinalis*).

Identification was based on standard morphological criteria according to established veterinary parasitology keys, according to Soulsby [17].

#### 2.3.6. Identification of Pulmonary and Larval Stages

Pulmonary strongylid larvae (L1) were recovered and identified based on morphological characteristics. *Dictyocaulus filaria* was confirmed by the presence of first-stage larvae with an anterior protoplasmic knob and dark granular intestinal inclusions [18]. Protostrongylid larvae (*Muellerius capillaris*, *Protostrongylus rufescens*) were differentiated based on characteristic tail morphology, particularly the structure of the caudal extremity [19].

### 2.4. Statistical Analysis

Statistical analyses were performed using GraphPad Prism version 5.0.0 (GraphPad Software, San Diego, CA, USA). The obtained data for both welfare indicators and endoparasitic infections were presented using two distinct but complementary metrics: prevalence rates (%) were calculated to show the frequency of affected animals within the population, while descriptive statistics (mean values, standard deviations, and standard error of the mean—SEM) were utilized to express the overall severity of welfare impairment and the quantitative intensity of parasitic infections. Data normality was assessed using the Kolmogorov–Smirnov test, which indicated that the variables were not normally distributed. Consequently, differences among welfare indicators were evaluated using the non-parametric Kruskal–Wallis test for median comparisons, with correction for tied ranks. Whenever significant differences were detected, Dunn–Bonferroni post hoc analysis was applied. The prevalence rates of both welfare indicators and endoparasitic infections (including coinfections) were calculated based on the total number of examined animals, and the statistical significance of differences between the two production systems was determined using the Chi-square (χ^2^) test. Relationships between the different welfare indicators and endoparasites were examined by Spearman’s rank correlation. For all correlation analyses, the absolute value of the Spearman’s correlation coefficients assessed whether weak (|r| < 0.30), moderate (0.30 ≤ |r| < 0.60), or strong (|r| ≥ 0.60) relationships existed, as described by Campbell [20]. In all statistical tests, values of *p* < 0.05 and *p* < 0.001 were considered the limit for statistical significance.

## 3. Results

### 3.1. Welfare Assessment

This study found that the welfare issues affecting ewes in confined production systems were thin body condition score, fleece cleanliness and quality, hoof overgrowth, udder asymmetry, and wool pulling. In pasture production systems, welfare issues were thin body condition score, fleece cleanliness, lesions, ocular and nasal discharges, respiration quality, fecal soiling, borderline anemia, and excessive itching. Significant variations in welfare indicators were observed between the two production systems (Table 1). Pasture production systems showed a significantly higher prevalence of welfare issues related to hygiene and health, such as fleece dirtiness, lesions, ocular and nasal discharges, respiration quality, fecal soiling, borderline anemia, and excessive itching (*p* < 0.05 to *p* < 0.001). Conversely, confined production systems were significantly more affected by management and behavior related indicators, including poorer fleece quality, hoof overgrowth, udder asymmetry, and stereotypic wool pulling (*p* < 0.05 to *p* < 0.001).

In sheep in pasture systems, the mean scores of thin BCS, fleece dirtiness, lesions (head, neck, legs, and udder), ocular and nasal discharges, respiration quality, fecal soiling, borderline anemia, and excessive itching were significantly higher than those for ewes in confined systems (*p* < 0.05, *p* < 0.001) (Table 2). The mean score of fleece quality, hoof overgrowth, udder asymmetry, and wool pulling—stereotypic behavior in confined systems were significantly higher than those for ewes in pasture systems (*p* < 0.01, *p* < 0.001) (Table 2).

### 3.2. Prevalence of Endoparasites

Overall, the prevalence of all identified endoparasites was significantly higher in the pasture management system compared to the confined system (*p* < 0.001, Table 3). *Eimeria* spp. and gastrointestinal strongyles were the predominant parasites across both groups, though their occurrence was markedly lower in confined housed ewes. Certain parasite species, including *Trichuris ovis* (4.94%), *Muellerius capillaris* (13.23%), *Dicrocoelium dendriticum* (53.97%), and *Giardia intestinalis* (1.94%), were recorded exclusively in the pasture management system (*p* < 0.001).

In contrast to other parasites, *Protostrongylus rufescens* was recorded exclusively in the confined management system (95% CI: 1.82–4.58) with a low prevalence (3.20%), while it was not diagnosed in the pasture system (*p* < 0.001). Cestodes of the *Moniezia* spp. were significantly more common in the pasture system (25.22%) than in the confined system (7.20%), which was confirmed by statistical analysis (*p* < 0.001) (Table 3).

The analysis of coinfections showed a significantly higher frequency of multiple infections in sheep from the pasture management system. Only double infections were recorded in both management systems, with a higher prevalence in the pasture system (20.81%) (*p* < 0.001). Multiple infections were diagnosed exclusively in the pasture system, with the highest prevalence of triple infections (34.74%). All statistical differences were highly significant (*p* < 0.001) (Table 3).

### 3.3. Intensity of Endoparasitic Infection

The production system significantly influenced the intensity of endoparasitic infections among the examined ewes (Table 4). In the confined production system, low-intensity infections predominated for both coccidia and gastrointestinal strongylids, while anoplocephalid infections were mostly of medium intensity. Conversely, ewes in the pasture production system exhibited a shift toward higher infection severities. On pasture farms, medium-intensity infections were the most frequent finding for gastrointestinal strongylids, with nearly a quarter of the positive animals developing high-intensity infections. Furthermore, while parasites such as *Trichuris ovis*, *Moniezia* spp., and various trematodes were present exclusively as low-intensity infections in the pasture system, *Giardia intestinalis* was predominantly recorded at a high intensity of infection (Table 4).

### 3.4. Correlations Between the Welfare Indicators and Endoparasites

Spearman’s rank correlation analysis revealed several significant relationships between parasite infections and welfare indicators (Table 5). In confined systems, weak but significant correlations were found between *Eimeria* spp. and thin BCS, as well as between gastrointestinal strongyles and both poor fleece quality and fecal soiling. In pasture systems, thin BCS, nasal discharge, and respiration quality were moderately correlated with *Muellerius capillaris* infection. Additionally, moderate correlations were noted between borderline anemia and *Dictyocaulus filaria*, and between fecal soiling and *Dicrocoelium dendriticum* (Table 5).

## 4. Discussion

The present results showed that the main welfare issues in ewes in confined and pasture systems were thin body condition score, fleece cleanliness and quality, lesions, ocular and nasal discharges, respiration quality, fecal soiling, hoof overgrowth, udder asymmetry, borderline anemia, excessive itching, wool pulling, and parasitological infections.

In the present study, a high proportion of ewes maintained an optimal BCS (81.44% in confined and 76.54% in pasture systems); however, the presence of thin ewes (17.76% and 21.34%, respectively) highlights a persistent factor that compromises welfare in those animals. This result is in line with those recorded by Stubsjøen et al. [21], who found that the majority of the ewes had a good BCS (70%), while 21% had a thin BCS. Also, Parés et al. [22] found that good body condition scores (BCSs) were higher in confined systems, indicating a more controlled nutritional management. Phythian et al. [23] highlighted that nutritional imbalances are common in pasture-managed livestock, severely compromising adult sheep welfare through nutrient deficiencies and subsequent metabolic disorders. In these pasture systems, where animals graze in open pastures, their nutritional intake relies heavily on the natural availability and quality of forage, which can vary greatly due to seasonal changes and climatic conditions [24]. In this study, a higher prevalence of thin ewes was observed in pasture compared to confined systems. This variance can be attributed to the energetic demands placed on free-ranging animals, which must travel significant distances daily to satisfy their nutritional requirements on pasture. This increased activity can greatly impact their maintenance requirements for metabolizable energy [25]. On the other hand, the observed thin BCS in ewes within confined systems may be due to food restriction or inadequate nutrition, which can negatively affect the animals’ affective state, even though sheep are adapted to low levels of nutrition [26]. In the present study, we found a statistically significant, weak correlation between thin BCS and *Eimeria* spp. in confined systems, and between thin BCS and *Muellerius capillaris* in pasture systems. It is well known that *Eimeria* species cause changes in the intestinal mucosa of infected animals, resulting in localized hemorrhage and atrophy that reduce intestinal absorption. This can lead to diarrhea, dehydration, anemia, limited growth and development, slowed weight gain, reduced feed utilization efficiency, exhaustion, and even death in severe cases in sheep [27,28]. Although coccidiosis typically affects young animals, this study found the presence of *Eimeria* spp. in ewes older than one year, which aligns with the findings of de Macedo et al. [29], who reported that animals of various ages are equally susceptible to *Eimeria* spp. In confined barn farming, high stocking densities and constant contact with bedding facilitate continuous, low-to-medium-intensity reinfections [30]. This persistent subclinical challenge triggers a localized intestinal inflammation that compromises the host’s energy balance over time, directly manifesting as a lower BCS and restricting nutrient availability for wool production and follicular health.

The results clearly confirm that the management system plays a crucial role in the epidemiology of endoparasitoses in sheep. The significantly higher prevalence of all identified parasites in the pasture management system compared to the confined system indicates that pasture conditions, continuous exposure to infectious stages, and the presence of intermediate hosts are the most important risk factors for the maintenance and spread of parasitic infections. No measures for the control of pathogens and parasites in the environment, including monitoring of drinking water quality, pasture management, or control of intermediate hosts, were implemented in the study area. It is well established that contaminated water sources and pastures represent important sources of infection with gastrointestinal helminths and protozoa, while the presence of dogs and wild canids may contribute to the maintenance of the life cycle of certain parasites, including species of the genus *Echinococcus*. Under grazing conditions, there is a potential risk of infection with this cestode in the investigated area, although the disease is generally asymptomatic in sheep. Therefore, measures such as pasture rotation, controlled grazing, regular removal of fecal contamination, provision of hygienically safe drinking water, and routine deworming of domestic and shepherd dogs are of particular importance for reducing infection pressure in pasture sheep production systems. Similar findings have been reported in recent studies from Europe, where extensive and semi-extensive management systems are consistently associated with a higher prevalence of gastrointestinal parasites in sheep [31]. The most prevalent parasite in this study was the *Eimeria* spp., with a very high prevalence in the pasture management system (83.60%), while the prevalence in the confined system was significantly lower (29.12%). These results are consistent with studies conducted in Italy, where authors reported a prevalence of *Eimeria* spp. of 86.36% on dairy sheep farms, with higher values recorded in extensive management conditions [32]. Additionally, research conducted in Spain and Portugal showed a prevalence of *Eimeria* spp. of 73.1% in sheep from extensive production systems [31]. The high frequency of coccidiosis in extensive systems can be attributed to the long survival of oocysts in the external environment, particularly in pastures with increased moisture and animal density. Furthermore, young animals in extensive farming often come into contact with large amounts of infectious material, which facilitates the rapid transmission of coccidia.

Gastrointestinal strongylids were the second most common group of parasites in this study, with a prevalence of 76.37% in the pasture management system and 26.40% in the confined system. These results align with data from numerous European studies that confirm the dominant role of gastrointestinal nematodes in grazing sheep. In Italy, gastrointestinal strongylids were significantly more frequently recorded on extensive sheep farms, with a prevalence of 54.55% [32], while in Germany, their prevalence in sheep was 62.9%, and in Spain and Portugal, it was 48.6% [33]. The higher prevalence observed in this study may be explained by climatic conditions favorable for the development of larval stages on pastures, as well as the potential emergence of resistance to anthelmintics, which has become increasingly common and is often reported in European countries. Research from Ireland indicates a significant rise in the resistance of gastrointestinal nematodes to multiple classes of anthelmintics, further complicating the control of these helminth infections in grazing sheep [34].

Notably, certain parasites (*T. ovis*, lung strongylids, trematodes, and *G. intestinalis*) were exclusively found in the pasture management system. Their absence in sheep from the confined system is predominantly linked to grazing conditions and contact with natural reservoirs of infection.

The finding of a high prevalence of *D. dendriticum* at 53.97% is significantly higher than the available data from most European countries. In Turkey, the prevalence of dicrocoeliosis was found to be 33.91%, while in Germany and the Iberian Peninsula, prevalences were recorded below 10% [35]. The elevated prevalence observed in this study may be linked to favorable agroecological conditions for the development of intermediate hosts (terrestrial snails and ants) and could also result from prolonged use of the same pastures without rotation and inadequate monitoring of infection status.

*Muellerius capillaris* was the most prevalent lung strongylid (13.23%) in this study, which aligns with data from other authors, indicating that this species is one of the most common protostrongylids in sheep across Europe [35]. The presence of lung nematodes almost exclusively in the pasture management system underscores the importance of intermediate hosts and grazing practices in their epidemiology. Interestingly, *P. rufescens* was recorded only in the confined management system, albeit with a low prevalence. This finding deviates from most literature, as infections with this species are typically associated with grazing systems. Specifically, since the confined farms in the Srem region utilize alfalfa hay harvested from local lowland fields, it is highly probable that land snails shedding *P. rufescens* larvae were accidentally collected during mechanical harvesting and subsequently ingested by the housed ewes through the contaminated forage.

*Giardia intestinalis* has been identified in sheep worldwide, with a global prevalence of 14–20% [36]. Although infections are mostly asymptomatic, they can lead to diarrhea, malabsorption, and reduced weight gain, particularly in lambs [37]. Given the zoonotic potential of this protozoan [36], the finding of nearly 2% in the examined sheep highlights the need for targeted, region-specific control strategies, especially in areas with confined farming practices, to prevent its spread. The occurrence of high-intensity *G. intestinalis* infection exclusively in the pasture production system suggests possible contamination of water sources or pastures.

Cestodes of the *Moniezia* spp. were significantly more common in the extensive management system, which is expected since infection occurs through the ingestion of infected free-living mites (family Oribatidae) present on pastures. A similar association between extensive farming and higher prevalence of monieziosis has been reported by authors from Spain and Portugal [31]. Although anoplocephalids are often considered parasites of lesser pathogenic significance, their presence indicates increased pasture contamination and the potential for concurrent infections with other helminths.

The results of the co-infection analysis indicate that multiple parasitic infections are a dominant characteristic of the pasture management system and were exclusively recorded in grazing sheep, while only dual infections were found in the confined system. These findings confirm contemporary research suggesting that co-infections are the norm rather than the exception in grazing ruminants [38]. In Germany, it was found that multiple infections are more prevalent in sheep than in cattle, with the most frequently diagnosed being dual infections with strongylids and *Eimeria* spp. [33]. Interactions between different parasitic species can lead to more pronounced clinical manifestations, reduced production performance, and further weakening of the host’s immune response. Crucially, overlapping symptoms in mixed infections such as diarrhea caused concurrently by *Eimeria* spp. and nematodes, or anemia linked to multiple helminth burdens, can mask the primary pathogen, potentially leading to clinical misdiagnosis and incorrect sheep treatments.

The results of the quantitative coprological analysis showed that the production system significantly affects both the prevalence and the intensity of infection with certain parasite species (gastrointestinal strongylids and *Eimeria* spp.), which were higher in the pasture production system. In the confined production system, clear differences were observed in the categories of low-intensity gastrointestinal strongylid infections and medium-intensity coccidial infections, alongside noticeable variations in low-intensity infections caused by *Moniezia* species. Conversely, the pasture production system showed distinct variations in both low- and high-intensity coccidial infections, with medium-intensity gastrointestinal strongylid infections being the most predominant. These findings indicate significant variations in the monitored quantitative parasitological parameters between different sheep production systems, reflecting a high level of pasture contamination and continuous exposure of animals to infective larval stages under grazing conditions. The detection of *T. ovis*, *Moniezia* spp., *D. dendriticum*, *F. hepatica*, and *Paramphistomum* spp. exclusively in the pasture production system, and solely as low-intensity infections, may indicate lower or limited exposure of sheep to these parasites, and further confirms the importance of management and grazing conditions in maintaining parasite transmission. The occurrence of high intensity *G. intestinalis* infection exclusively in the pasture production system suggests possible contamination of water sources or pastures.

Wool cleanliness acts as an indicator of the sheep’s environment and overall management practices. Clean wool suggests that the sheep are kept in a well-maintained habitat with proper hygiene, while dirty wool may reflect poor environmental conditions, such as muddy or wet areas, and inadequate care [11]. In present study, the prevalence of ewes with wet, soiled fleece with mud or feces was significantly higher in pasture (60.14%) compared with confined systems (26.40%). The possible reasons for this condition in pasture systems could be influenced by environmental factors such as mud, water, and vegetation, which contribute to wool soiling, particularly during wet conditions [39,40]. This was especially relevant during the study period, which took place in autumn and winter, when such environmental factors were prevalent. In confined systems, factors such as overcrowding, poor hygiene, and inadequate bedding management practices can lead to higher levels of fecal and urine contamination [21]. Additionally, nutritional deficiencies and health issues, such as parasitic infections, can also impact wool cleanliness in both systems [21]. According to Færevik et al. [41], sheep prefer to rest on soft, dry surfaces, and the comfort they experience, both physical and mental, is influenced by the type and quantity of flooring materials available. Being forced to lie on wet or dirty ground can negatively affect their overall comfort levels.

In this study, the prevalence of lesions on the head, neck, legs, and udder was significantly higher in pasture compared with confined systems. These results are in agreement with those recorded by Parés et al. [22], who mainly observed lesions in flocks with access to pasture. This finding in pasture systems could be the result of the foraging behavior of sheep, which primarily rely on grazing. However, when food resources are scarce, they may resort to seeking nourishment from shrubs and other vegetation, leading to lesions and fleece loss [40]. Additionally, lesions on sheep can occur due to ectoparasites such as sheep lice, sheep scab, sheep ked, and the sheep blowflies that irritate the skin [42], and traumas, the type and quality of the equipment used during handling or housing, conflicts with other sheep, and interactions with other animals [11].

Ocular discharges were significantly more prevalent in ewes in pasture systems compared to those in confined systems. This condition in pasture systems may be influenced by environmental factors, such as strong winds and heavy snowfall. According to Hosie [43], infectious keratoconjunctivitis is often associated with cold and harsh weather.

In the present study, we found significantly more ewes with nasal discharges and respiratory problems in pasture systems compared with confined systems. These results are in agreement with those recorded by Parés et al. [22], who found that respiratory symptoms tended to be higher on semi-extensive farms than on semi-intensive and intensive farms. The available literature suggests that different factors influence nasal discharges and respiratory problems in sheep, such as infectious diseases (viral or bacterial), internal parasites, poor environmental conditions, allergies, nutritional deficiencies, and stress [44,45,46]. The results also showed correlations with *Muellerius capillaris* and nasal discharges, and *Muellerius capillaris* and respiratory problems. These results can be ascribed to the fact that the most common clinical signs of lungworm *Muellerius capillaris* in sheep are moderate-to-persistent coughing, rapid or labored breathing (dyspnea), nasal discharge, loss of appetite, and weight loss [46]. The damp pastures of Fruška Gora Mountain favor the survival of terrestrial gastropods, the essential intermediate hosts for *Muellerius capillaris*. As these nematodes mature, they induce chronic interstitial pneumonia [46], significantly increasing the animals’ respiratory workload. For pasture-managed ewes that must walk long distances across hilly terrain to graze, compromised pulmonary gas exchange, combined with increased physical exertion, severely drains energy reserves, directly correlating with lower body condition scores.

Wool is essential for sheep, as it provides insulation, helping to regulate body temperature and protect against environmental elements like rain and ultraviolet (UV) radiation. It also aids in preventing skin injuries and infections, while serving as a valuable economic resource in textiles and other industries [47]. In this study, some fleece loss and significant fleece loss in ewes were more prevalent in confined systems compared with pasture systems. Wool loss could be the result of many factors, such as higher stress levels due to overcrowding and less space; a deficiency of roughage, copper, zinc, cobalt, calcium, phosphorus, sodium chloride, and manganese in the diet; poor nutrition during pregnancy; lameness and increased plasma cortisol; and various microbial infections and parasites [39,48,49]. In confined systems, we found a moderate significant correlation between fleece quality and Strongilidae, and fleece quality and *Eimeria* spp., while in pasture systems, we found a weak correlation between fleece quality and *Eimeria* spp. This may reflect indirect effects of chronic parasitism on nutritional status and immunity, predisposing animals to fleece loss from other causes. It is widely recognized that infections with GIN reduce the efficiency of dietary protein use, which affects tissue growth, bone development, and wool production in sheep [6].

Although mastitis and lameness were observed in very low proportions in confined and pasture systems, they are significant welfare concerns due to the pain and discomfort which reduces mobility, feed intake and weight gain, decreasing body condition and wool production [50]. The results for lameness in this study are lower than those reported by Stubsjøen et al. [21], who observed 0.4% of animals as lame, but higher than the findings of Phythian et al. [23], who indicated a mean lameness prevalence of 7.1% in sheep farms in England and Wales. Hoof overgrowth was significantly prevalent in confined systems (20.96%) compared with pasture systems (1.94%). These results can be ascribed to the fact that in confined sheep production systems, hoof overgrowth is more common because sheep move less and their hooves do not wear down naturally. Wet and dirty ground conditions limit natural abrasion, making hooves more susceptible to overgrowth and subsequent lameness. Nutritional factors, such as diets that are high in energy but low in fiber, can also affect hoof growth and health [51].

Fecal soiling was significantly prevalent in ewes in pasture systems (16.23%) compared with confined systems (5.44%). According to Dwyer et al. [11], fecal soiling refers to the accumulation on fecal material around the anus, tail, and hind legs, usually caused by diarrhea due to parasites or poor nutrition, and it may lead to flystrike. The results also showed a weak significant correlation between Strongilidae and fecal soiling in confined systems, and a weak and moderately significant correlation between *Eimeria* spp. and fecal soiling, and between *Dicrocoelium dendriticum* and fecal soiling in pasture systems. These findings may be explained by the pathogenic effects of *Dicrocoelium dendriticum*, which can cause diarrhea, weight loss, submandibular edema, and, in severe cases, death of the animals [7]. In addition, gastrointestinal nematode infections are widely recognized as important risk factors for diarrhea in sheep [52].

Anemia is defined by a hemoglobin level that falls below a certain limit, which can differ depending on the species and the animal’s physiological state. According to Albers et al. [53], in sheep, significant anemia is frequently caused by infections with gastrointestinal nematodes, particularly *Haemonchus contortus*, an abomasal blood feeder. In this study, we found significant differences between borderline anemia in sheep in confined and pasture systems, with the higher prevalence in pasture systems (16.23%). Also, we found weak and moderate correlations between borderline anemia and *Trichuris ovis*, and between borderline anemia and *Dictyocaulus filaria* in pasture systems. A possible explanation for the association between *Trichuris ovis* and borderline anemia in sheep is that the parasite causes mild intestinal inflammation and blood loss, which can lead to decreased red blood cell levels and contribute to anemia, especially in cases of high parasite burden or other health issues [54]. Additionally, metabolites and host–parasite interaction products of *Dictyocaulus filaria* may have a detrimental effect on circulating erythrocytes, increasing their fragility and potentially further contributing to the development of anemia [55].

Excessive itching is considered abnormal behavior and not part of normal grooming; rather, it suggests the presence of ectoparasites [11]. In our research, we observed that only ewes in pasture systems manifest excessive itching. Previous studies have shown that the major ectoparasites affecting small ruminants include mites, lice, ticks, and fly larvae that attack the skin, wool, or hair of animals and can cause disease as well as severe discomfort, irritation, itching, rubbing, biting, and depressed wool growth in sheep [39,56]. The presence of ectoparasites may have contributed to the excessive itching issue in the ewes, despite not being examined.

In our results, we recorded wool puling only in ewes in confined systems. This result is in line with findings reported by other researchers who found that only indoor sheep show this abnormal behavior [22,57]. Many factors could affect wool pulling, such as a restrictive environment [58], husbandry deficiencies [59], and being fed with diets of small particle size, which do not stimulate rumination [60]. According to Cockram [61], abnormal behavioral responses in sheep in confined systems could be a serious animal welfare problem that is difficult to control.

### Study Limitations

Although this study provides comprehensive insights into the relationship between management systems, welfare indicators, and endoparasite prevalence, several methodological limitations should be acknowledged. First, the cross-sectional design and the specific sampling period from November to April restrict our ability to evaluate seasonal dynamics, as parasite shedding and certain weather dependent welfare issues (e.g., fleece cleanliness or respiratory problems) vary significantly during the warmer summer months. Second, because our primary focus was on comparing the two production systems, data were aggregated across farms without accounting for the hierarchical nesting of animals within individual units, which represents a clustering effect that may mask farm management variations. Third, due to the production cycles within this selected period, the ewes were evaluated across mixed physiological stages (late gestation, lactation, and post-weaning), which influence metabolic demands, immunity, and susceptibility to parasitic burdens. Fourth, while visual assessments of hoof overgrowth were strictly conducted without manual restraint to eliminate acute handling stress, this approach did not allow for a detailed clinical examination of pododermatitis or interdigital lesions. Lastly, parasite identification relied on morphological and quantitative coprological analyses; incorporating molecular diagnostic tools (e.g., PCR) would enhance species-level identification, particularly for strongylid and *Eimeria* species, while specific diagnostic screenings for ectoparasites could further clarify the exact causes behind indicators like excessive itching. Additionally, post-mortem or serological evaluations would be required to assess internal tissue-dwelling zoonoses, such as *Echinococcus granulosus*, which cannot be detected via standard coprological screening.

## 5. Conclusions

Based on the welfare indicators and parasitological analysis, ewe welfare is significantly compromised in both production systems, though the underlying causes and manifestations distinctively differ. The confined system is primarily associated with production, morphological, and behavioral issues (poorer fleece quality, hoof overgrowth, udder asymmetry, and stereotypic wool pulling). To mitigate these challenges, confined management must implement regular, scheduled hoof trimming to counteract the lack of natural flooring wear, optimize stocking densities, and increase dietary roughage to stimulate rumination and reduce abnormal behaviors. Conversely, the pasture system is primarily challenged by health and hygiene-related issues, dominated by a high prevalence of individual and mixed endoparasitic infections (primarily *Eimeria* spp., gastrointestinal strongyles, *Dicrocoelium dendriticum*, and *Muellerius capillaris*), with specific parasitic burdens directly correlating with thin body condition score, fleece cleanliness, ocular/nasal discharges, respiratory problems, fecal soiling, and borderline anemia. Addressing these pasture-specific risks requires establishing veterinary protocols, including continuous coprological monitoring and strategic pasture rotation, alongside providing adequate shelter or windbreaks to protect animals from harsh weather elements. Continuous monitoring and system specific management adjustments remain essential for maintaining optimal sheep health and welfare across both production systems.

## Figures and Tables

**Table 1:** Prevalence of welfare indicators in confined and pasture production systems.

Welfare Indicators			Confined Systems (n = 625)		Pasture Systems (n = 567)		χ^2^	*p*-Value
			N	% (CI95%)	N	% (CI95%)		
Body Condition Score	Emaciated		5	0.80 (0.10–1.50)	12	2.12 (0.93–3.31)	3.67	0.06
	Thin		111	17.76 (14.76–20.76)	121	21.34 (17.97–24.71)	2.43	0.11
	Good		509	81.44 (72.52–85.36)	434	76.54 (70.24–82.84)	4.31	0.04
Fleece cleanliness	Wet, soiled with mud or feces		165	26.40 (22.99–29.81)	341	60.14 (56.12–64.16)	138.54	0.0001
	Very wet, heavily soiled		9	1.44 (0.52–2.36)	24	4.23 (2.57–5.89)	8.61	0.003
Lesions	Head		4	0.64 (0.05–1.23)	13	2.29 (1.07–3.51)	5.78	0.02
	Neck		1	0.16 (0–0.47)	23	4.06 (2.45–5.67)	22.88	0.0001
	Legs		8	1.28 (0.45–2.11)	28	4.94 (3.18–6.70)	13.58	0.0001
	Udder		4	0.64 (0.05–1.23)	27	4.76 (3.03–6.49)	19.94	0.0001
Ocular discharge			13	2.08 (1.05–3.11)	36	6.35 (4.37–8.33)	13.75	0.0001
Nasal discharge			34	5.44 (3.73–7.15)	177	31.22 (27.44–35.00)	135.60	0.0001
Respiration quality			27	4.32 (2.80–5.84)	95	16.75 (13.65–19.85)	50.04	0.0001
Fleece quality		Some fleece loss	225	36.00 (32.26–39.74)	84	14.81 (11.89–17.73)	69.48	0.0001
		Significant fleece loss	38	6.08 (4.14–8.02)	20	3.53 (2.01–5.05)	4.19	0.04
Mastitis			0	0	3	0.53 (0–1.12)	3.32	0.07
Lameness			17	2.72 (1.55–3.89)	19	3.35 (1.88–4.82)	0.40	0.53
Fecal soiling		Soiling and dags	34	5.44 (3.66–7.22)	92	16.23 (13.17–19.29)	36.58	0.0001
		Extensive soiling and dags	5	0.80 (0.58–1.02)	26	4.59 (2.87–6.31)	16.82	0.0001
Hoof overgrowth			131	20.96 (17.75–24.17)	11	1.94 (0.82–3.06)	102.49	0.0001
Udder asymmetry			9	1.44 (0.56–2.32)	0	0	8.23	0.004
Excessive itching			0	0	92	16.23 (13.17–19.29)	109.89	0.0001
Borderline anemia			20	3.20 (1.91–4.49)	92	16.23 (13.17–19.29)	59.25	0.0001
Wool pulling			52	8.32 (6.24–10.40)	0	0	49.33	0.0001

N—total number of samples; n—number of positive samples; χ^2^—Chi-squared test value; Significant differences are indicated by *p* < 0.05.

**Table 2:** Mean scores of welfare indicators across different production systems.

Welfare Indicators		Confined Systems	Pasture Systems	*p*-Value
		Mean (±SD)		
Body Condition Score		2.81 ± 0.42	2.74 ± 0.48	0.04
Fleece cleanliness		0.29 ± 0.49	1.33 ± 1.01	0.0001
Lesion	head	0.01 ± 0.08	0.02 ± 0.15	0.02
	neck	0.002 ± 0.04	0.04 ± 0.20	0.0001
	legs	0.01 ± 0.0.11	0.05 ± 0.22	0.0001
	udder	0.01 ± 0.08	0.05 ± 0.21	0.0001
Ocular discharge		0.02 ± 0.14	0.06 ± 0.24	0.0008
Nasal discharge		0.05 ± 0.23	0.31 ± 0.46	0.0001
Respiration quality		0.04 ± 0.20	0.17 ± 0.37	0.0001
Fleece quality		0.48 ± 0.61	0.22 ± 0.49	0.0001
Mastitis		0	0.01 ± 0.07	0.06
Lameness		0.03 ± 0.16	0.03 ± 0.18	0.53
Fecal soiling		0.19 ± 0.76	0.67 ± 1.32	0.0001
Hoof overgrowth		0.21 ± 0.41	0.02 ± 0.14	0.0001
Udder asymmetry		0.01 ± 0.12	0	0.002
Excessive itching		0	0.15 ± 0.35	0.0001
Borderline anemia		0.03 ± 0.18	0.16 ± 0.37	0.0001
Wool pulling		0.08 ± 0.27	0	0.002

SD—standard deviation; exact *p*-values are displayed in the final column, with *p* < 0.05 considered statistically significant.

**Table 3:** Prevalence of endoparasites and coinfections in examined ewes.

Endoparasites	Confined Systems (n = 625)		Pasture Systems (n = 567)		χ^2^
	N	% (CI95%)	N	% (CI95%)	
*Eimeria* spp.	182	29.12 (25.56–32.68)	474	83.60 (80.55–86.65)	356.54
Gastrointestinal strongyles	165	26.40 (22.94–29.86)	433	76.37 (72.87–79.87)	296.02
*Trichuris ovis*	0	0	28	4.94 (3.16–6.72)	31.61
*Dictyocaulus filaria*	0	0	20	3.53 (2.01–5.05)	22.42
*Muellerius capillaris*	0	0	75	13.23 (10.44–16.02)	88.22
*Protostrongylus rufescens*	20	3.20 (1.82–4.58)	0	0	18.45
*Moniezia* spp.	45	7.20 (5.17–9.23)	143	25.22 (21.65–28.79)	72.67
*Dicrocoelium dendriticum*	0	0	306	53.97 (49.87–58.07)	453.01
*Fasciola hepatica*	0	0	28	4.94 (3.16–6.72)	31.61
*Paramphistomum* spp.	0	0	40	7.05 (4.94–9.16)	45.62
*Giardia intestinalis*	0	0	11	1.94 (0.80–3.08)	12.24
**Coinfections**					
Two infections	53	8.48 (6.29–10.67)	118	20.81 (17.47–24.15)	36.79
Three infections	0	0	197	34.74 (30.82–38.66)	260.15
Four infections	0	0	146	25.75 (22.15–29.35)	183.40
Five infections	0	0	10	1.76 (0.68–2.84)	11.12
Six infections	0	0	10	1.76 (0.68–2.84)	11.12

N—total number of samples; n—number of positive samples; χ^2^—Chi-squared test value; all listed comparisons between confined and pasture production systems are statistically significant at the *p* < 0.001 level; CI—confidence interval.

**Table 4:** Quantitative assessment of fecal samples across different production systems.

Degree of Infection (Quantitative FEC Method)		Endoparasites							
		E	GIS	TO	M	DD	FH	P	GI
**Confined systems**									
	**N**	**182**	**165**	**/**	**45**	**/**	**/**	**/**	**/**
**Low**	n	112	142	/	12	/	/	/	/
	%	61.54	86.06	/	26.67	/	/	/	/
	Mean ± SEM	142.90 ± 9.08	204.90 ± 9.44	/	375 ± 7.54	/	/	/	/
**Medium**	n	42	23	**/**	33	**/**	**/**	**/**	**/**
	%	23.08	13.94	/	73.33	/	/	/	/
	Mean ± SEM	1193 ± 33.15	906.30 ± 28.28	/	816.70 ± 26.08	/	/	/	/
**High**	n	28	/	/	/	/	/	/	/
	%	15.38	/	/	/	/	/	/	/
	Mean ± SEM	1952 ± 54.16	/	/	/	/	/	/	/
**Pasture systems**									
	**N**	**474**	**433**	**28**	**143**	**306**	**28**	**40**	11
**Low**	n	238	92	28	143	306	28	40	/
	%	50.21	21.25	100	100	100	100	100	/
	Mean ± SEM	228 ± 9.06	77.72 ± 6.20	89.28 ± 8.28	236.40 ± 13.06	196.10 ± 8.16	133.90 ± 8.93	95 ± 11.57	/
**Medium**	n	203	236	**/**	**/**	**/**	**/**	**/**	4
	%	42.83	54.50	/	/	/	/	/	36.36
	Mean ± SEM	965.50 ± 20.18	1023 ± 25.57	/	/	/	/	/	1175 ± 72.17
**High**	n	33	105	/	/	/	/	/	7
	%	6.96	24.25	/	/	/	/	/	63.64
	Mean ± SEM	2591 ± 122.5	2458 ± 92.60	/	/	/	/	/	2164 ± 219.80

E—*Eimeria* spp.; GIS—gastrointestinal strongyles; TO—*Trichuris ovis*; M—*Moniezia* spp.; DD—*Dicrocoelium dendriticum*; FH—*Fasiciola hepatica*; P—*Paramphistomum* spp.; GI—*Giardia intestinalis*; low: <50–500 opg cpg/epg; medium: 550–1500 opg/cpg/epg; high: >1500 opg/cpg/epg (opg/epg, number of oocysts/cysts/eggs calculated per 1 g of feces); N—total number of samples; n—number of positive samples.

**Table 5:** Spearman’s rank correlations between welfare indicators and endoparasites.

Endoparasites	Welfare Indicators	r	*p*-Value
**Confined systems**			
*Eimeria* spp.	Thin BCS	0.20	0.003 **
Strongilidae	Fleece quality	0.20	0.0001 ***
*Eimeria* spp.	Fleece quality	0.15	0.0001 ***
Strongilidae	Fecal soiling	0.24	0.0001 ***
**Pasture systems**			
*Muellerius capillaris*	Thin BCS	0.30	0.0001 ***
*Muellerius capillaris*	Nasal discharge	0.48	0.0001 ***
*Muellerius capillaris*	Respiration quality	0.54	0.0001 ***
*Trichuris ovis*	Borderline anemia	0.21	0.0001 ***
*Dictyocaulus filaria*	Borderline anemia	0.31	0.0001 ***
*Eimeria* spp.	Fecal soiling	0.23	0.0001 ***
*Dicrocoelium dendriticum*	Fecal soiling	0.32	0.0001 ***

***—*p* < 0.001; **—*p* < 0.01; r—Spearman’s rank correlation coefficient.

## Data Availability

The original contributions presented in this study are included in the article. Further inquiries can be directed to the corresponding author.
